# Identification of CTLA-4 associated with tumor microenvironment and competing interactions in triple negative breast cancer by co-expression network analysis

**DOI:** 10.7150/jca.46301

**Published:** 2020-09-09

**Authors:** Ziqi Peng, Peng Su, Yuhong Yang, Xue Yao, Yiqi Zhang, Feng Jin, Bowen Yang

**Affiliations:** 1 Department of Breast Surgery, the First Affiliated Hospital of China Medical University, Shenyang, China.; 2 Department of Medical Oncology, the First Hospital of China Medical University, Shenyang, China.; 3 Medical Record Management Center, the First Hospital of China Medical University, Shenyang, China.; 4 Disease prevention and infection control Office, Liaoning Cancer hospital & Institute, Shenyang, Liaoning Province, China.; 5 Department of Surgical Oncology, the First Hospital of China Medical University, Shenyang, China.

**Keywords:** CTLA-4, Immune, TNBC, hsa-mir-92a, WGCNA

## Abstract

**Background:** The study of CTLA-4 inhibitors has been one of the hot spots in the field of tumor immunotherapy. As the most immunogenic subtype of breast cancer, Triple negative breast cancer (TNBC) has a great potential in the treatment strategy. The aim of this study was to explore the relevant genes and pathways of CTLA-4 in TNBC and to explore the prognostic value, so as to provide a theoretical basis for clinical studies.

**Materials and methods:** We used the data from The Cancer Genome Atlas (TCGA) to analyze the expression of CTLA-4 in different types of breast cancer, and analyzed the TNBC data of CTLA-4 related co-expression genes by WGCNA and enrichment analysis. LncRNA-miRNA-CTLA-4 network was constructed to explore the immune infiltration and immune checkpoint associated with CTLA-4. The effect of CTLA-4 on clinical outcomes in TNBC patients was also evaluated. Finally, we used data from GEO database to verify the differences of CTLA-4 in different molecular types of breast cancer and related prognostic results.

**Results:** CTLA-4 was significantly higher in TNBC than in Luminal subtype and Her-2 + subtype (*P=*0.019 and *P<*0.001, separately), and was significantly higher in ER and PR negative samples than in ER and PR positive samples (*P<*0.001). CTLA-4 related genes mainly enriched in biological process of leukocyte differentiation, regulation of leukocyte activation and T cell activation. Hsa-mir-92a was found to be a survival significance marker associated with CTLA-4 and lncRNA-miRNA-CTLA-4 network was constructed. The results of immune infiltration analysis showed that CTLA-4 was mainly related with T cell (*r=*0.74). For immune checkpoints analysis, CTLA-4 was mainly related to PDCD1(*r=*0.72) and CD28(*r=*0.64). In TNBC, high expression of CTLA-4 is related to good survival (*P=*0.0061). Results consistent with previous analysis were obtained in the GEO database, the expression of CTLA-4 in TNBC was significantly higher than that in non-TNBC (*p<*0.001), CTLA-4 was associated with favorable survival of TNBC (*p<*0.001).

**Conclusion:** Among all types of breast cancer, the expression of CTLA-4 was the highest in TNBC.CTLA-4 in TNBC can be regulated by hsa-mir-92a to form ceRNA networks and influence the prognosis of TNBC patients through the leukocyte differentiation, regulation of leukocyte activation and T cell activation pathway.

## Introduction

Breast cancer is the most common malignant tumor in women and its mortality rate ranks the first among female with an increasing trend 1. Since breast cancer is a highly heterogeneous tumor, there are great differences in molecular profiles, cell types and biological behavior. This has led to differences in the response and clinical outcomes of patients to conventional treatments, including chemotherapy, endocrine therapy, radiotherapy and targeted therapy 2.

Among all types of breast cancer, triple negative breast cancer (TNBC) is a type of breast cancer with highly malignancy, poor prognosis and high risk of recurrence and metastasis 3, accounting for about 15-20 percent of all breast cancer patients 4. Since the expressions of estrogen receptor (ER), progesterone hormone receptor (PR) and human epidermal growth factor receptor 2 (HER-2) were poor, the lack of effective treatment made it very troublesome. However, with the development of immunotherapy strategies in the field of breast cancer, there seems to be a new idea for this intractable problem. The study showed that, comparing with non-TNBC, there were significant enrichment of immune activity and immune pathway in TNBC. Among the BC subtypes, TNBC showed the strongest immunogenicity 5.This also indicated that immunotherapy has great potential in TNBC treatment strategies.

In recent years, the clinical application of immune checkpoint molecular antibody has brought hope to tumor immunotherapy. Immune checkpoint molecules are protective molecules in human immune system. Tumor cells can against human immune response by overexpressing immune checkpoint molecules and relevant ligands, evade immune surveillance and immune killing, and promote self-growth. Currently, immunization checkpoints have been reported mainly as follows: CTLA-4, PD-1, TIGIT, TIM-3, LAG-3 and VISTA 6. Among them, CTLA-4, as the first immune checkpoint discovered, it's a leukocyte differentiation antigen which is the transmembrane receptor of T cells. It shares the B7 with CD28 and binds to B7 to maintain T cell activity and participates in negative regulation of immune response 7. CTLA-4 antibody induces anti-tumor immunity by blocking Foxp3+Treg cells in the tumor microenvironment and AKT phosphorylation pathway, effectively amplifying T cells and enhancing the anti-tumor effect 8.

Currently, CTLA-4 inhibitors mainly include Tremelimumab and Ipilimumab, which have been successively approved by the FDA for the treatment of melanoma, non-small cell lung cancer, advanced kidney cancer and other tumors 9-11. However, no CTLA-4 inhibitor has been approved for the treatment of breast cancer. The first immunosuppressant approved for the treatment of breast cancer, the PD-L1 inhibitor (Atezolizumab), was released in 2019, and it was approved by the FDA to be used in combination with Abraxane in patients with inoperable, locally advanced or metastatic triple-negative breast cancer 12. One study showed that the CTLA-4 inhibitor combined with Exemestane stabilized 11 of 26 patients with ER+/ HER2-breast cancer for 12 weeks or longer, with an optimal ORR of 42% 13. In a study of the safety and tolerance of Ipilimumab mediated immune regulation in breast cancer, preoperative tumor cryoablation combined with Ipilimumab showed induction and synergistic antitumor effects 14. Scientists studying the family of melanoma antigens found that CTLA-4 inhibitors enable TNBC patients with MAGE-A (melanoma associated antigen A) expression to achieve a greater immune response during treatment 15. These results suggest that CTLA-4 is of great value in the TNBC immunotherapy.

**Figure 1:**
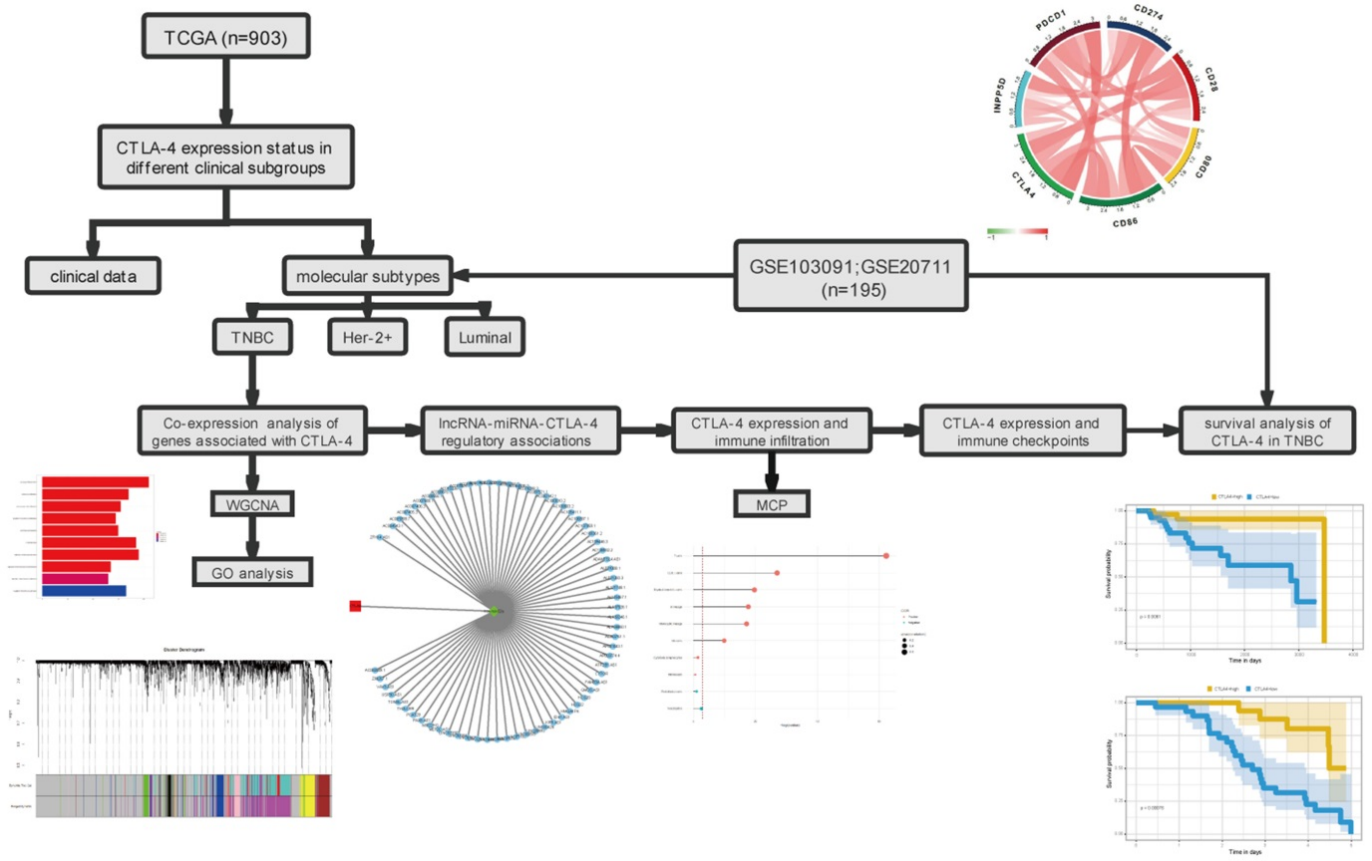
The workflow of the study.

However, comparing with other tumors, studies on CTLA-4 in breast cancer are still immature. Currently, studies are mostly in phase I and phase II clinical trials. We believe that a comprehensive analysis of CTLA-4 in breast cancer is necessary in order to explore its potential for use alone or in combination with other therapies. In this study, we analyzed the expression of CTLA-4 in different types of breast cancer, and then selected TNBC to comprehensively analyze the expression of CTLA related genes. The association of CTLA-4 with other immune checkpoints and immune infiltration were also evaluated. Further analysis showed that CTLA-4 can be regulated by hsa-mir-92a, forming the regulatory mechanism of ceRNA network and affecting the prognosis of TNBC patients. These results suggesting that CTLA-4 could be used as a prognostic marker of TNBC and it will provide a good theoretical basis for future clinical trials. The flowchart of this study is shown in Fig. 1.

## Materials and Methods

### Study design and patients

RNA-Seq data and corresponding clinical data were downloaded from the TCGA data portal (https://portal.gdc.cancer.gov/). All included sample data downloaded from TCGA meet the following criteria: (a) Patients with mRNA, lncRNA and miRNA expression and clinical data (b) Patients diagnosed with breast cancer by pathology before June 2018 (c) All the treatment plans of the patients were formulated under the guidance of the guidelines and they all had completed adjuvant therapy as planned after surgery. (d) Patients had survival information and the survival time was greater than 10 days. TCGA data were used for all analysis related to CTLA-4. Another independent validation data set was obtained from the Gene Expression Omnibus (GEO) database under the accession number GSE103091 (https://www.ncbi.nlm.nih.gov/geo/query/acc.cgi?acc=GSE103091) and GSE20711 (https://www.ncbi.nlm.nih.gov/geo/query/acc.cgi?acc=GSE20711). The inclusion criteria for GEO datasets are as follows: (a) The dataset can be used to determine TNBC patients (b) Complete clinical prognostic indicators are available (c) TNBC samples were more than 10. These data were used to verify the differential expression of CTLA-4 in different molecular types of breast cancer and the associated prognosis.

### Definition of TNBC-related genes by WGCNA and enrichment analysis

WGCNA (weighted gene co-expression network analysis) is a method to construct gene co-expression network based on gene expression data. It is more accurate to obtain co-expressed genes by WGCNA, we searched the co-expressed genes for CTLA-4 in TNBC by using this method. First, we chose the top 5000 genes after sorting by mean absolute deviation (MAD) and the R package of WGCNA was used to construct the co-expression network for mRNA expression of the above genes. Then we chose a soft-thresholding parameter β to build a proximity matrix that matches our gene distribution to a scale-free network based on connectivity. After that, the adjacency was transformed into topological overlap matrix (TOM), and the linkage hierarchical clustering was carried out on average according to the dissimilarity measure based on TOM. Finally, we chose a genes dendrogram with a minimum (genome) of 30 and a module dendrogram with a cut-line of 0.25, and combined some modules to produce more rigorous results. ClusterProfiler is an R package for enrichment analysis 16. After co-expression analysis, we use it to analyze the biological process of the related genes in the Gene Ontology (GO) 17.

### LncRNA-miRNA-CTLA-4 regulatory associations

In order to determine the miRNA and lncRNA related to CTLA-4, we used Pearson analysis to find the miRNA negatively related to CTLA-4 in TNBC. Then, we found miRNAs that regulate CTLA-4 expression on the miRwalk (http://mirwalk.umm.uni-heidelberg.de/) platform. We intersected the two results to obtain the survival significance of miRNA regulating CTLA-4 gene expression in TNBC. Then, we used Pearson analysis to find lncRNAs negatively correlated with miRNA expression in TNBC data. Finally, multi-type RNA signature was constructed as the linear combination of expression values of CTLA-4 related miRNAs, and lncRNAs.

### Association between immune factors and CTLA-4

Tumor invasive immune cells (TIC) participate in the formation of tumor immune microenvironment, regulate tumor growth, and affect the survival of patients and the efficacy of anti-tumor therapy. MCP-counter is an R package able to quantify 8 immune cell species and 2 stromal cell populations in heterogeneous tissues based on expression data 18. It generated CD3+ T cells, CD8+ T cells, cytotoxic lymphocytes, NK cells, B lymphocytes for each sample, derived from monocytes (monocyte lineage), myeloid dendritic cells, neutrophils, and endothelial and fibroblast abundance scores. Then we used correlation analysis to assess the relationship between CTLA-4 and immune infiltration in TNBC. In addition, we also evaluate the correlation between CTLA-4 and immune checkpoints in TNBC.

### Relationship between CTLA-4 expression and survival in TNBC

To determine the relationship between CTLA-4 expression and survival in TNBC that significantly correlated with survival, we used TCGA RNA-seq expression profile data to perform Kaplan-Meier (KM) survival analysis of CTLA-4 and used GEO data to verify the results.

### Statistical analysis

The statistical analysis was mainly processed by using R language (https://www.r-project.org/) with a few public packages available. We use T test to analyze the different expression of CTLA-4 in different clinical groups. The lncRNA-miRNA-target regulatory network was constructed by using cytoscape software. The correlation between CTLA-4 expression and immune infiltration and immune checkpoints in TNBC were explored by using pearson correlation analysis. Kaplan-Meier method based on log-rank test was used to generate the survival curve. Other figures were processed by several R packages, such as WGCNA, corrplot and circlize. The BH method was used to correct all multiple tests. *P* value < 0.05 was considered to be statistically significant.

## Results

### Data downloading and collection

A total of 903 breast cancer patients with mRNA expression from TCGA datasets were included in this study, with an average age of 58.63 years and a standard deviation of 13.2. Among them, there were 726 luminal patients, 33 Her-2 + patients and 144 TNBC patients. The other part of the validation set from the GEO database, we chose GSE103091 and GSE20711 and they included 195 breast cancer patients, with an average age of 56.52 years and a standard deviation of 12.19. Among them, there were 107 TNBC patients and 88 non-TNBC patients. The relevant patient information was shown in Table S1 and Table S2.

### Expression status of CTLA-4 in different clinical subgroups

**Figure 2:**
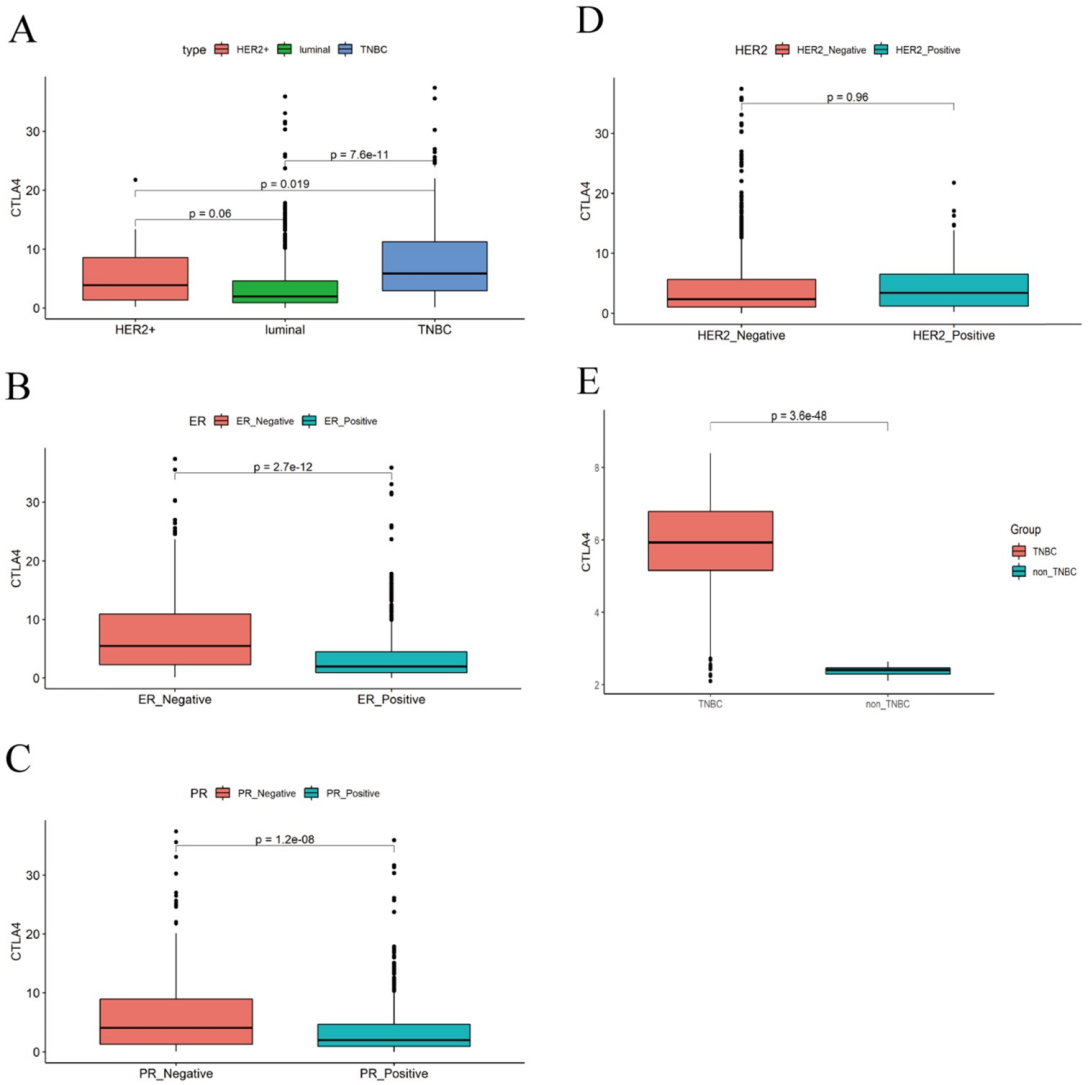
** Differences in CTLA-4 expression between different clinical subgroups. A.** CTLA-4 expression in different molecular type. **B-D.** CTLA-4 expression in ER/PR/Her-2 positive or negative. **E.** The expression of CTLA-4 in different molecular types was verified by GEO data.

We used TCGA data to analyze the level of CTLA-4 in different clinical subgroups. As the fig.2A shows, the expression of CTLA-4 in TNBC is significantly higher than in Luminal subtype (*p=*0.019) and Her-2+ subtype (*P<*0.001). The difference of CTLA-4 expression was not statistically significant between Luminal subtype and Her-2+ subtype (*P=*0.06). As shown in Fig. 2B-D, the level of CTLA-4 in patients with negative ER expression was significantly higher than that in patients with positive ER expression (*P<*0.001). Similarly, CTLA-4 expression was significantly higher in patients with negative PR expression than in patients with positive PR expression (*P<*0.001). CTLA-4 showed no statistically significant difference between patients with positive Her-2 expression and those with negative Her-2 expression (*P=*0.96).

### Co-expression network construction

**Figure 3:**
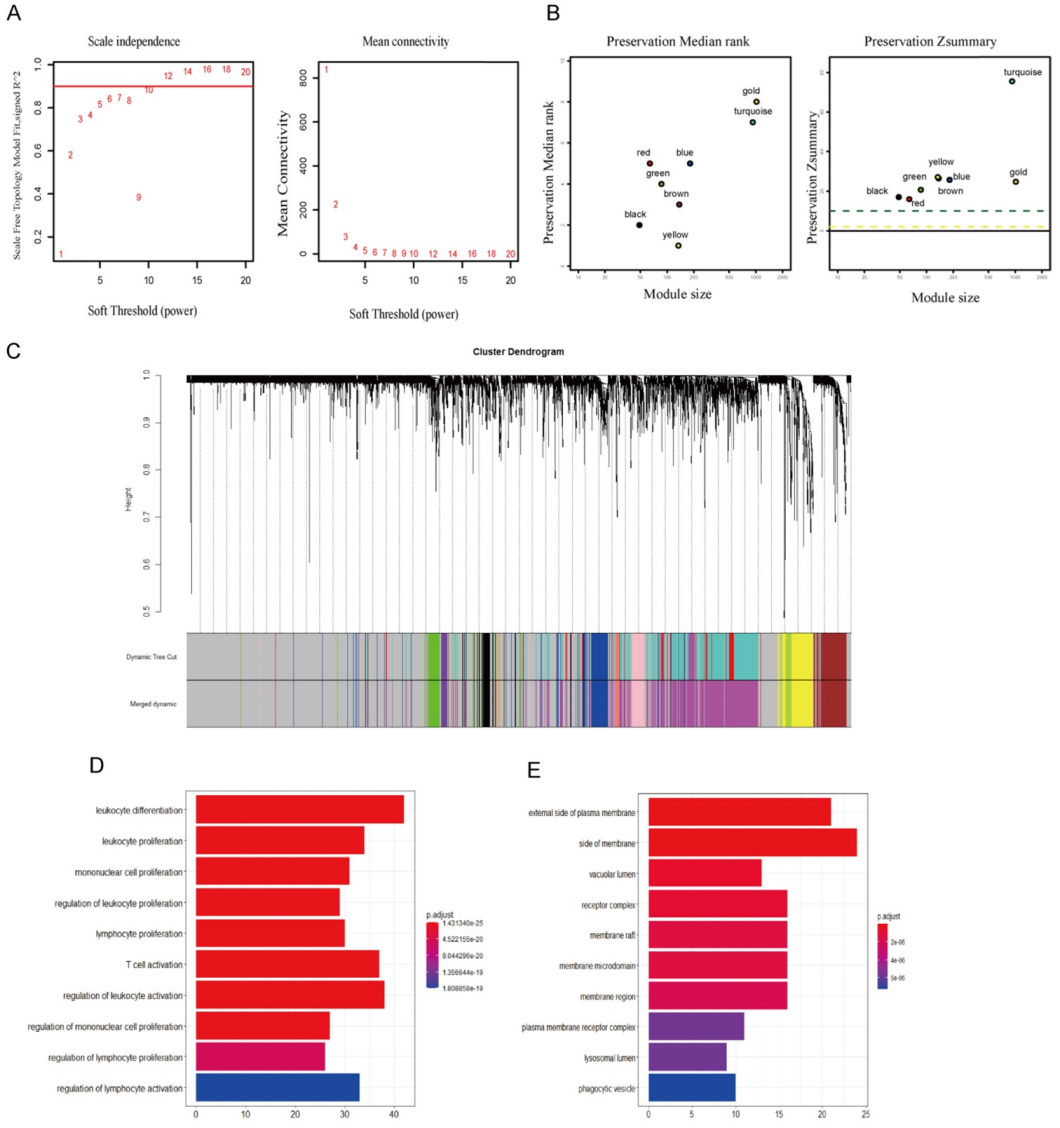
** Co-expression analysis of genes associated with CTLA-4. A.** Soft threshold selection in the WGCNA network analysis. **B.** The medianRank and Zsummary statistics of the module preservation. **C.** Gene distribution in the WGCNA network analysis. **D-E.** GO analysis for the CTLA-4 co-expression genes.

We analyzed the data with WGCNA to identify the modules containing highly correlated genes with CTLA-4 in TNBC and the analysis included 144 TNBC samples. First, we found that when we set the soft threshold to 12, it conformed to the scale-free property of biological network, so we used β=12 to construct the weighted network (Fig. 3A), Second, average linkage hierarchical clustering, the TOM-based dissimilarity, dynamic tree clipping, and merging processing were used to identify modules, We obtained a total of 11 meaningful modules marked with different colors, among which the gene co-expressed with CTLA-4 belongs to the yellow module (Fig. 3B-C). After that, 199 genes co-expressed with CTLA-4 obtained by WGCNA were used to make an enrichment analysis. CTLA-4 related genes mainly enriched in biological process of leukocyte differentiation, regulation of leukocyte activation and T cell activation (Fig. 3D and Table 1) and in cellular component of external side of plasma membrane and side of membrane (Fig. 3E).

### LncRNA-miRNA-CTLA-4 regulatory network

**Figure 4:**
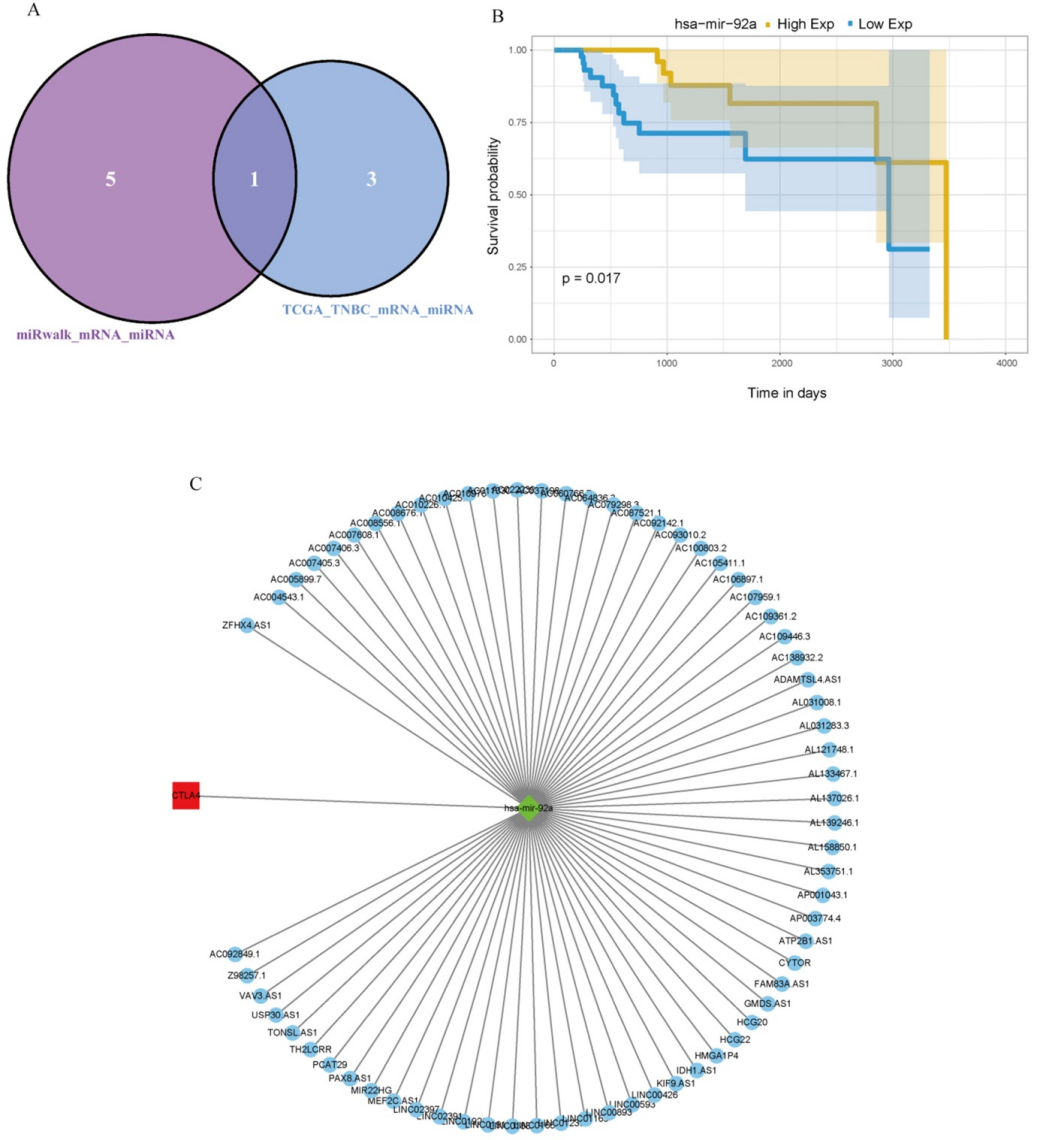
** lncRNA-miRNA-CTLA-4 regulatory associations. A.** The Venn diagram was used to screen the central miRNA between TCGA data and wiRwalk. **B.** Prognostic analysis of has-mir-92a in TNBC. **C.** The lncRNAs-miRNAs-CTLA-4 regulatory network.

First, Pearson analysis was used to analyze the TPM of different miRNAs and lncRNAs in TNBC, and four miRNAs negatively correlated with the expression of CTLA-4 were found. Then, six miRNAs that could regulate CTLA-4 were analyzed on the miRwalk platform and after taking the intersection of the two results, hsa-mir-92a was obtained (Fig. 4A). It was verified that hsa-mir-92a had survival significance in TNBC (*p=*0.017, Fig. 4B). Then we analyzed 68 lncRNAs negatively correlated with the expression of hsa-mir-92a in TNBC data. Finally, we made lncRNA-miRNA-target regulatory network by using cytoscape, as shown in Fig. 4C.

### CTLA-4 expression and immune infiltration

**Figure 5:**
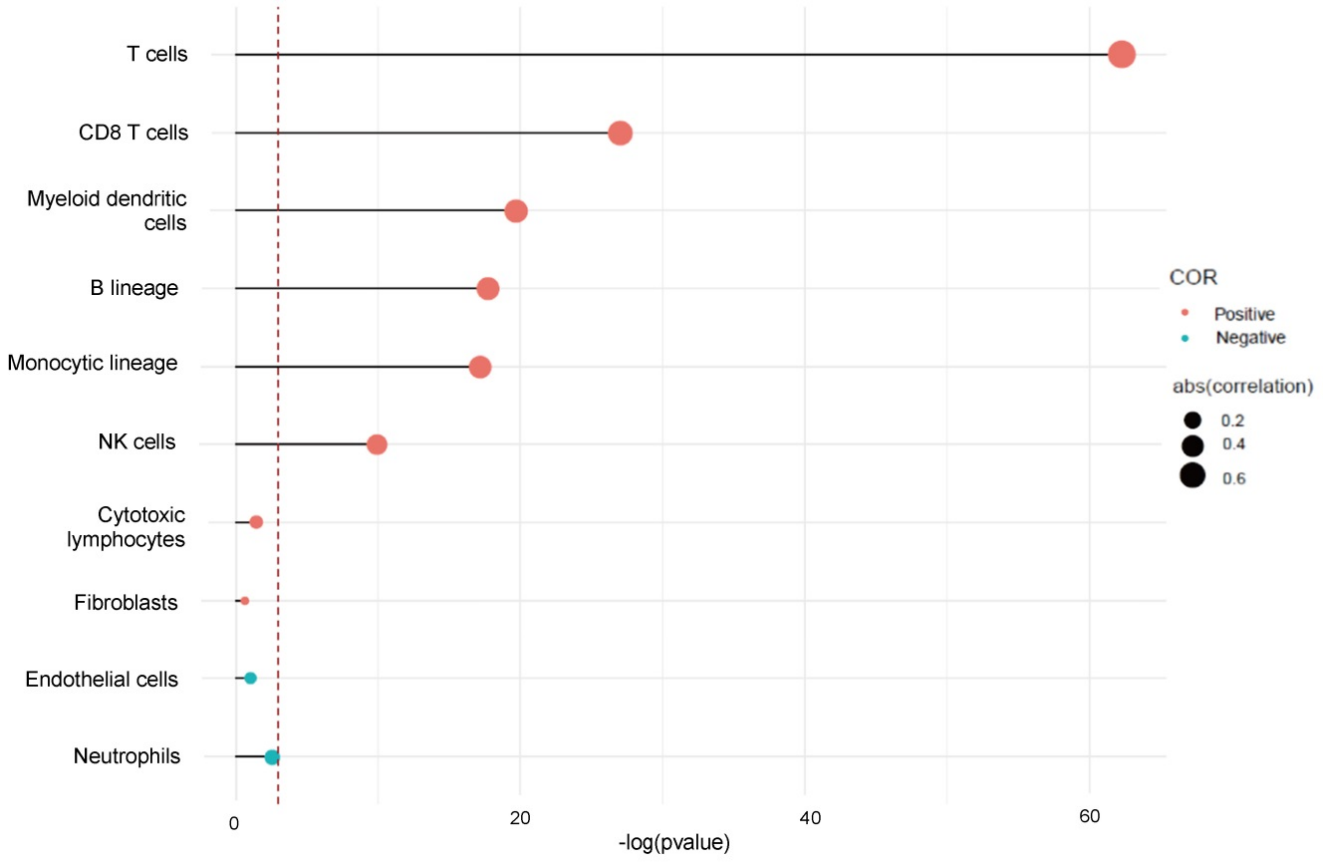
CTLA-4 expression and immune infiltration.

We used Microenvironment Cell Population-counter to analyze the profiles of immune infiltration in TNBC. We also analyzed the populations of immune cells associated with CTLA-4 in TNBC. The results showed that CTLA-4 was mainly related with T cell (*r=*0.74) (Fig. 5).

### CTLA-4 expression and immune checkpoints

**Figure 6:**
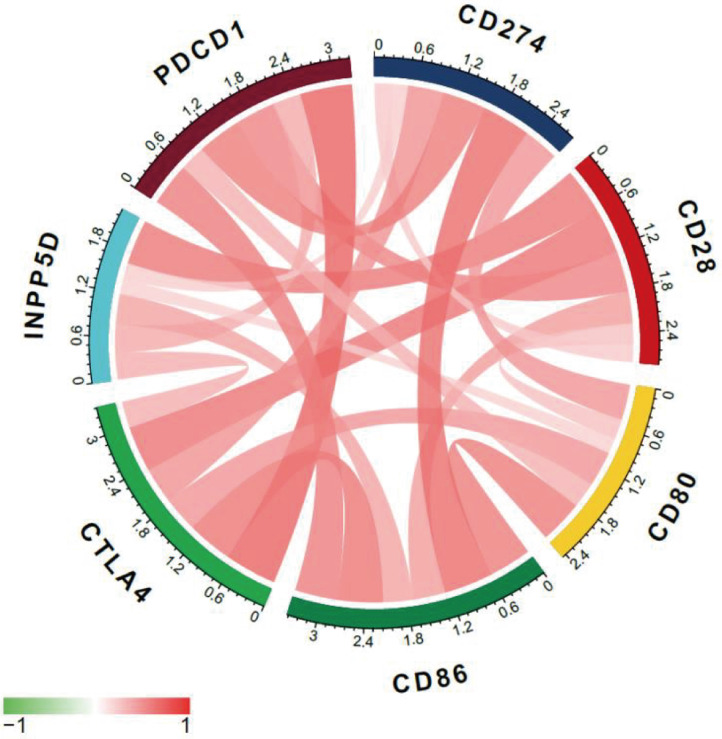
Co-expression analysis between CTLA-4 and immune checkpoints.

Multi-immune checkpoint therapy is expected to be a new trend of tumor immunotherapy 19. Here we analyzed the relevance of expression between CTLA-4 and other crucial immune checkpoints, such as CD28, CD86, CD80, and so on. As shown in Fig. 6 and Table 2, CTLA-4 was mainly related to PDCD1 (*r=*0.72) and CD28 (*r=*0.64).

### Survival analysis

**Figure 7:**
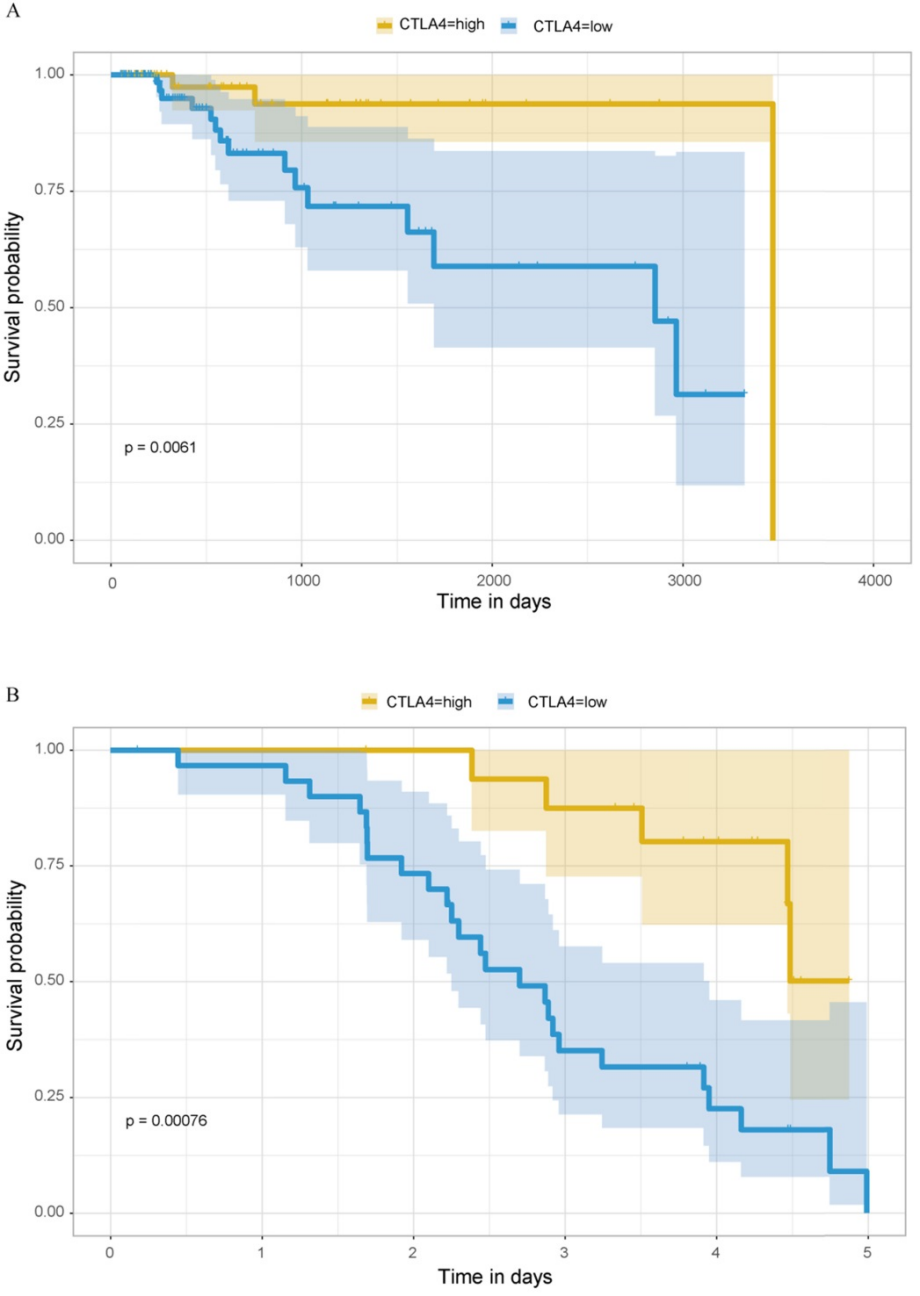
** Prognostic analysis of CTLA-4 in TNBC. A.** Prognostic analysis in TCGA database. **B.** Prognostic analysis in GEO database.

Previous analyses have concluded that TNBC, as the type of breast cancer with the worst prognosis, has significantly higher expression of CTLA-4 than other molecular types of breast cancer. Then we explored the relationship between CTLA-4 expression and prognosis in TNBC. As shown in Fig. 7A, it is worth considering that in TNBC, high level of CTLA-4 is related to good survival (*P=*0.0061).

### GEO database validation

We verified the expression difference of CTLA-4 in TNBC and non-TNBC by using 195 samples from GEO database, and obtained the same results as before. The expression of CTLA-4 in TNBC was significantly higher than that in non-TNBC (Fig. 2E, *p<*0.001). At the same time, we also verified the relationship between the expression of CTLA-4 in TNBC and survival, and obtained the same results as before (Fig. 7B, *p<*0.001).

## Discussion

Immunotherapy has attracted extensive attention because of its innovative and excellent efficacy. Whether it is chemotherapy or radiotherapy, it will damage patients' bodies and reduce immunity while killing cancer cells. It has been examined that expression of CTLA-4 is associated with age, with increased expression in elderly breast cancer 20. The researchers studied five related gene models of CTLA-4 and found that gene polymorphism is associated with breast cancer susceptibility, such as CTLA-4 +6230G > A can effectively reduce the risk of homozygous and recessive breast cancer in the Chinese population 21. In general, TNBC has abundant support of tumor infiltrating lymphocytes and heterogeneous tumor microenvironment, and has long been considered as a potential protagonist in the immunotherapy of breast cancer, but TNBC itself also has strong heterogeneity 22. Therefore, we need to comprehensively analyze the regulatory mechanism of CTLA-4 in TNBC, so as to provide a basis for clinical individualized treatment.

Using data from TCGA database and GEO database to analyze the expression of CTLA-4 among different molecular subtypes, we concluded that the expression of CTLA-4 in TNBC was significantly higher than that in other molecular subtypes of breast cancer. By analyzing the expression of CTLA-4 at different ER, PR, Her-2 expression status in different breast cancer samples, we concluded that the expression of CTLA-4 was higher in the samples of breast cancer patients with negative estrogen receptor expression, while the expression of CTLA-4 was not statistically significant at different Her-2 expression levels. Therefore, from the conclusion that CTLA-4 is highly expressed in TNBC, we speculate that compared with other types of breast cancer, CTLA-4 has potential value in exploring immunotherapy for TNBC.

Further we made a comprehensive analysis of CTLA-4 related gene in TNBC and made an enrichment analysis. Previous studies have shown that CTLA-4 is only expressed on T cells and can maintain its own tolerance by inhibiting the activation and proliferation of T cells, thus protecting the body from the effects of autoimmunity 23. In one study, CTLA-4 expression and T cell activation were studied in 1,087 breast cancer patients. The results showed that CTLA-4 expression was associated with T cell activation, with high CTLA-4 expression corresponding to low T cell activation score, which was negatively correlated with survival rate 24. This indicates that it is of great significance for us to analyze the biological process of CTLA-4-related genes. At the same time, we screened out a miRNA, hsa-mir-92a, that could regulate CTLA-4 and had survival significance in TNBC, and we predicted 68 lncRNAs that could regulate hsa-mir-92a as well. There have been studies in cervical and ovarian cancers, the report said that the occurrence of cervical cancer is related to the up-regulation of hsa-mir-92a and the serum hsa-mir-92 can be used as an independent indicator of cervical cancer 25. Hsa-mir-92a can also inhibit peritoneal proliferation of ovarian cancer cells by inhibiting the expression of integrin α5 26. However, hsa-mir-92a has not been reported in breast cancer.In our study, we hypothesized that CTLA-4 in TNBC can be regulated by hsa-mir-92a which forms ceRNA network through the above biological pathways and affects the prognosis of TNBC patients. We evaluated the status of immune infiltration in TNBC and analyzed the number of CTLA-4-related immune cells in TNBC. The results showed that CTLA-4 had the strongest correlation with T lymphocytes. In breast cancer patients, TILs (tumor infiltrating lymphocytes) is considered as one of the indicators to evaluate prognosis and guide treatment.Studies have shown that the number of TILs in breast cancer with different molecular typing is different, and the response to immunotherapy is also different. There are more TILs in HER-2 positive type and TNBC, and the number of TILs is positively correlated with disease-free survival and other prognostic indicators 27, 28. Studies have also confirmed that in TNBC, the more TILs, the better the effect of immunotherapy is 29. Host microenvironment, especially immune infiltration, is an important factor in predicting immune checkpoint inhibitor response. This suggests the importance of exploring the relationship between CTLA-4 and immune infiltrating cells.

We then examined the correlation between CTLA-4 and some common immune checkpoints and CTLA-4 was found mainly related to PDCD1 and CD28.CTLA-4 shares the B7 molecular ligand with CD28, and is negatively regulated by the immune response 7. PDCD1 (PD-1) is an important immunosuppressive molecule. PD-1 and PD-L1 combining initiated programmed T cell death, enabling tumor cells to obtain immune escape 30. Moreover, previous studies have found that the expression level of PD-1 / PD-L1 in TNBC is higher than that of other types of breast cancer, and its expression level is positively correlated with the malignant degree of TNBC 31. PD-1 and CTLA-4 have non-redundant functions in inducing and maintaining immune cell activation. A combination therapy that blocks these two immune checkpoints is more effective than a single immune checkpoint inhibitor was found in seven tumors and this combination therapy have been approved for clinical use. A phase I trial of TNBC showed that PD-1 and CTLA-4 antibodies can be used in combination to inhibit the growth of triple negative breast tumors carrying BRCA1 mutations 32. In this study, the level of CTLA-4 in TNBC was strongly correlated with the expression of PD-1, which also provides further theoretical support for this treatment strategy.

Finally, we performed prognostic analysis in TCGA data and verified with sample information in GEO database, and obtained a result worthy of consideration. The higher the level of CTLA-4, the better the prognosis of TNBC patients is. In previous studies on tumors, high expression of CTLA-4 always associated with poor prognosis. Based on reports of all types of breast cancer, CTLA-4 is an independent predictor of shorter DFS and OS in breast cancer 33. It has also concluded that breast cancer patients with high CTLA-4 expression have a significant tendency to metastasize to the axillary lymph nodes 34. The higher the density of CTLA-4 in lymphocytes, the lower the expression in breast cancer cells, and the better the prognosis will be 35.However, the relationship between CTLA-4 expression in tumor cells of TNBC patients and prognosis has been rarely reported. This study shows that high level of CTLA-4 in TNBC is good prognosis, and the underlying mechanism is worth exploring.

So far, studies on CTLA-4 inhibitors have been very limited. Compared with other tumors, studies on CTLA-4 inhibitors in breast cancer are still immature. However, according to the existing research results, whether CTLA-4 inhibitor is a single drug, or it is combined with other immune checkpoint inhibitors, combined with chemotherapy or combined with radiotherapy, it has their potential value. In this paper, a comprehensive analysis of CTLA-4 was made and a biomarker has-mir-92a was proposed, which can provide a greater possibility for further exploration of its utilization value.

## Supplementary Material

Supplementary table S1.

## Figures and Tables

**Table 1:** Top ten terms of GO analysis for CTLA-4

Gene	Description	GENERATIO	*P* value	*P* adjust	Count
CTLA-4	leukocyte differentiation	42/168	8.51E-29	1.43E-25	42
	leukocyte proliferation	34/168	1.02E-28	1.43E-25	34
	Mononuclear cell proliferation	31/168	3.26E-26	3.04E-23	31
	Regulation of leukocyte proliferation	29/168	1.78E-25	1.25E-22	29
	lymphocyte proliferation	30/168	4.66E-25	2.61E-22	30
	T cell activation	37/168	9.01E-25	4.20E-22	37
	regulation of leukocyte activation	38/168	3.23E-24	1.29E-21	38
	regulation of mononuclear cell proliferation	27/168	1.03E-23	3.62E-21	27
	regulation of lymphocyte proliferation	26/168	1.74E-22	5.43E-20	26
	regulation of lymphocyte activation	33/168	6.64E-22	1.81E-19	33

**Table 2:** Co-expression analysis between CTLA-4 and immune checkpoints

Gene	CTLA-4_R	CTLA-4_P
CD28	0.640168138	1.49542E-18
INPP5D	0.371343914	3.12429E-06
PDCD1	0.723787193	1.85477E-25
CD86	0.632366738	5.15697E-18
CD80	0.493936757	1.54075E-10
CD274	0.501988554	6.92903E-11

## Abbreviations

TNBC: Triple negative breast cancer
TCGA: The Cancer Genome Atlas TCGA
lncRNA: long non-coding RNA
ceRNA: competing endogenous RNA
ER: estrogen receptor
PR: progesterone hormone receptor
HER2: human epidermal growth factor receptor 2
WGCNA: weighted gene co-expression network analysis
TIC: tumor invasive immune cells
TILs: tumor infiltrating lymphocytes
