# Supplementary material for: Identification of CTLA-4 associated with tumor microenvironment and competing interactions in triple negative breast cancer by co-expression network analysis
Source: J Cancer. 2020 Sep 9;11(21):6365–75. doi: 10.7150/jca.46301 (PMC7532512; doi:10.7150/jca.46301)
Supplement: Supplementary file 1 — Supplementary table S1. [file jcav11p6365s1.pdf]

Table S1 Clinical features of 903 breast cancer patients in TCGA database

Molecular type	n	Age(mean)	Age(sd)	percent
Luminal	726	59.33	13.46	80.4%
Her-2+	33	58.24	13.37	3.7%
TNBC	144	55.18	12.04	15.9%
total	903	58.63	13.2	100%

Table S2 Clinical features of 195 breast cancer patients in GEO database

Molecular type	n	Age(mean)	Age(sd)	percent
TNBC(GSE103091)	107	56.97	12.68	54.9%
Non-TNBC(GSE20711)	88	55.97	11.46	45.1%
total	195	56.52	12.19	100%
